# Multiple regulatory aspects of histone methyltransferase EZH2 in Pb-induced neurotoxicity

**DOI:** 10.18632/oncotarget.19615

**Published:** 2017-07-27

**Authors:** Wei-Zhen Xue, Xiaozhen Gu, Yulan Wu, Danyang Li, Yi Xu, Hui-Li Wang

**Affiliations:** ^1^ School of Food Science and Engineering, Hefei University of Technology, Hefei, Anhui 230009, PR China

**Keywords:** Pb, neurotoxicity, EZH2, chromatin immunoprecipitation, PRC2

## Abstract

Pb is a pervasive environmental threat to human health. Although remarkable progress has been made in its neurotoxicity, the precise molecular mechanisms underlying this widespread toxicant still remain elusive. In this study, the detailed roles of EZH2, a transcriptional repressor, in the regulation of Pb-led neurotoxicity were investigated, highlighting its sub-functionalization, compartmentalization, functional chaperones and downstream partners. Based on the findings, EZH2’s protein levels were significantly reduced in response to Pb treatment; EZH2’s gain-of-function trials recovered the dampened neurite outgrowth; EZH2’ recruitment to ploycomb complex, as well as its interaction with cytosolic Vav1, was altered in a distinct manner, suggesting that EZH2’s multiple roles were markedly redistributed in this context; EZH2’s cytosolic and nuclear presence differed in their respective response towards Pb treatment; EZH2 directly occupied the promoters of *EGR2*, *NGFR* and *CaMKK2*, genes responsible for various nerve functions and repair mechanisms, and essentially contributed to their aberrant expression. It indicated that EZH2 mediated the dynamic changes of a cascade of key molecules and consequently the related neurological impairments. In summary, EZH2 emerges as a central player to regulate Pb-led neurotoxicity in a transcriptionally dependent and independent manner, and thereby provided a promising molecular target for medical intervention.

## INTRODUCTION

Pb is a pervasive environmental threat that continues to compromise human health on a global scale [1, 2]. Despite its ubiquitous presence, Pb is a chemically xenobiotic element with no known essential function in cellular growth, proliferation or physiological function [3, 4]. Pb almost pervades in every organ of human body, but central nervous system (CNS) has long been recognized as the primary target of its toxicity [5]. Childhood Pb exposure could cause a range of intellectual and behavioral deficits on children, such as impaired cognition function, Intelligence Quotient (IQ), grade performance, etc [4, 6, 7]. To date, mounting evidence showed that Pb exposure was linked with the development of some neurological disorders, manifested by hyperactivity disorder, depression, Alzheimer’s and Parkinson’s diseases [5].

Accumulating data have suggested the Pb’s detrimental effect on children cognition and behavior at blood levels less than 5 μg/dl, thereby leading to a widely-accepted notion: no level of Pb appears to be safe [3, 8]. The exact mechanisms underlying low-level Pb exposure and its neurotoxicity were not well understood. And several modes of actions have been proposed: oxidative stress, membrane-biophysics alteration, dysregulation of signaling pathway and impairment of neurotransmission. Intriguingly, Chetty et al. [9] assumed that the mechanisms of Pb-led neurotoxicity were largely divergent, and could be classified as morphological, neurochemical and stressed ones. Specifically, the detailed molecular targets and their interactions involved in this complex intoxication are warranted to be further established.

The enhancer of zeste homolog 2 (EZH2), a transcriptional repressor, is a catalytic subunit of the Polycomb repressive complex 2 (PRC2) and induces the trimethylation of the histone H3 lysine 27 (H3K27me3) [10, 11]. EZH2 is an evolutionarily conserved protein, and numerous studies have highlighted its roles in fine-tuning cellular differentiation. In addition, EZH2 is also strongly linked with the maintenance of cell identity, cell cycle regulation, and particularly cancer biology being currently at the cutting edge of the research [12–14]. *EZH2*’s identity as oncogene has attracted intense interest, with its aberrant expression levels (mostly elevated) intrinsically associated with tumor progression and poor prognosis [15]. Meanwhile, EZH2 could also act as a tumor suppressor, since acquired EZH2 mutations in myeloid neoplasms have been identified [16].

EZH2 was also implicated in the proper function of central nervous system (CNS). It was evidenced [17] that knocking down EZH2 augmented the intracellular Ca^2+^ level, thereby enhancing neurogenesis of mesenchymal stem cells. Strikingly, the methyltransferase activity of EZH2 was required to maintain the balance between neural stem cell proliferation and differentiation into neurons, or in other instances, into oligodendrocytes [18]. Due to its critical involvement, EZH2’s abnormal performance was also a strong indicator of several neurological disorders [18]. To date, EZH2 has not been associated with the environmental etiology of the neurological diseases. Collectively, considering its key roles in CNS’s function, whether EZH2 is mechanistically involved in Pb-led neurotoxicity, remains an open question.

Pb intoxication did not possess a particular “behavioral signature”, thus leading to diverse outcomes in nervous system [19]. In order to identify and characterize the “molecular signatures” involved in this process, we focused on EZH2’s roles in regulating Pb-led neurotoxicity in PC 12 cells, a rat pheochromocytoma, one of the most widely studied cell line for neurotoxicity studies [7]. We specifically examined its detailed behavioral patterns, highlighting its sub-functionalization, compartmentalization, functional chaperones and downstream genes. Consequently, EZH2 was suggested as a central player governing the entire neurotoxic process, shedding light on the mechanistic studies of Pb-led neurotoxicity.

## RESULTS

### EZH2’s expression is inhibited by Pb exposure in a dose- and time-dependent manner

**Figure 1:**
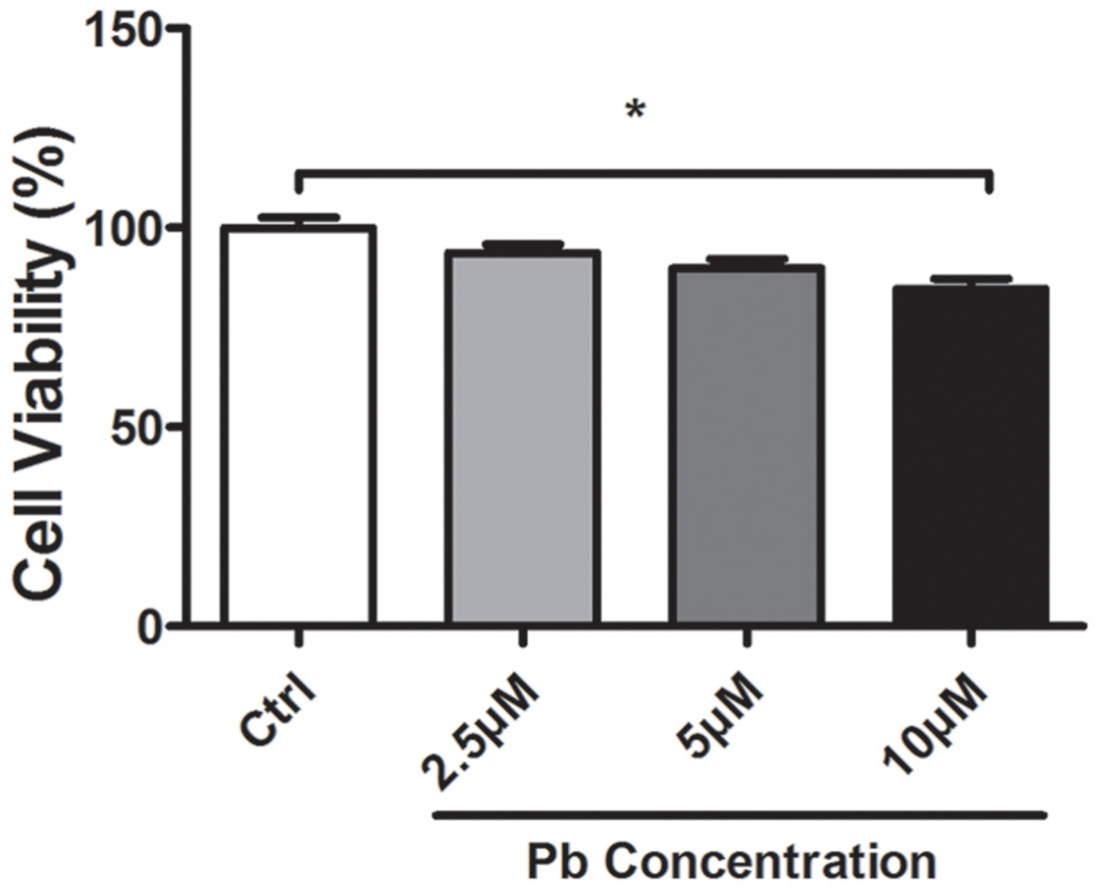
Cell viability altered by Pb exposure with various concentrations for 24 h The data was obtained from the MTT reduction assay on PC 12 cells. Values represent mean ± SEM of triplicate experiments. * Indicates significantly different (*P*<0.05) between the indicated groups.

Cell viability of Pb exposure with varying doses was assessed via the MTT reduction assay. It was shown from Figure 1 that, a treatment with 10 μM Pb significantly compromised the PC 12 cells’ viability, wherein a lower dose did not yield a lethal effect. To exclude the mechanistic complexity brought by cell death, Pb exposure with 5 μM concentration was primarily utilized in the following investigations. A low-level Pb exposure also better favored the elucidation of the genuine neurotoxic effect caused by this xenobiotic metal.

**Figure 2:**
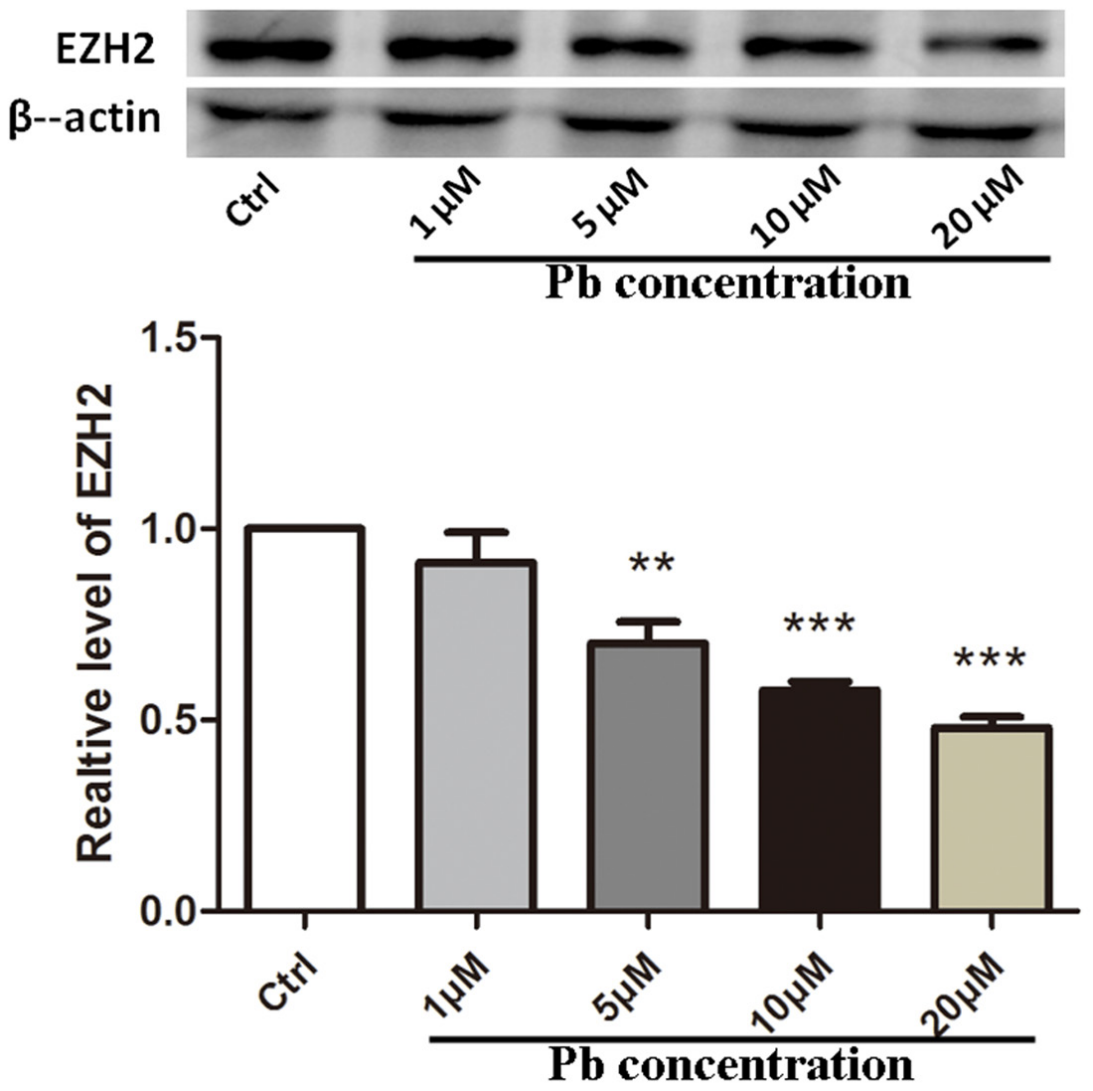
EZH2’s protein expression altered by Pb exposure with various concentrations for 24 h on PC 12 cells Quantification of western blotting results for EZH2 was shown in the lower portion. The maximum value obtained from the control sample was set as “1” for normalization. Data were shown as mean ± SEM (n=3). ** and *** indicated significantly different (*P*<0.01 and *P*<0.001) compared to control, respectively. Representative western blots of EZH2 and β-actin (internal control) proteins were shown in the upper portion.

To gain a knowledge of EZH2’s roles in Pb-led neurotoxicity, its protein levels are first required to be examined. As evidenced by Western blotting (WB) analysis (Figure 2), EZH2 levels were markedly decreased due to the administration of Pb into PC 12 cells (with normalized data as 0.7009±0.05584 and 0.5777±0.02231 for 5μM and 10μM, respectively). Otherwise, EZH2’s expression was further inhibited along with the increment of toxicant’s dosage (within the scope of 20 μM, 0.4792±0.02922), suggesting a linear dose-response relationship. The concerted alterations implied an involvement of EZH2 in this neurotoxic process.

**Figure 3:**
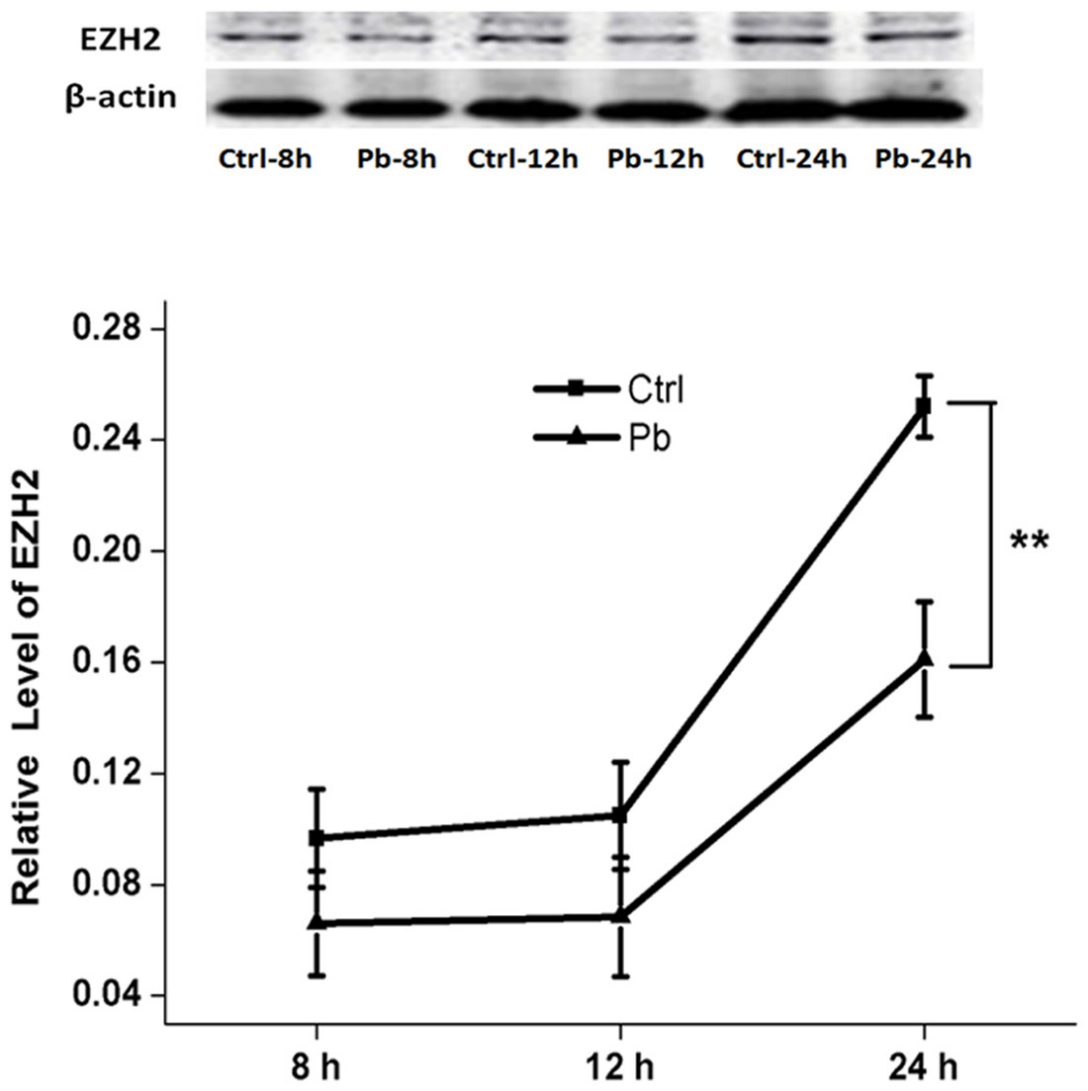
EZH2’s protein expression altered by 5 μM’s Pb exposure with various incubation periods on PC 12 cells Quantification of western blotting results for EZH2 was shown in the lower portion. The protein level of EZH2 was calculated as compared to the respective control group. Data were shown as mean ± SEM (n=3). ** indicated significantly different (*P*<0.01). Representative western blots of EZH2 and β-actin (internal control) proteins were shown in the upper portion.

**Figure 4:**
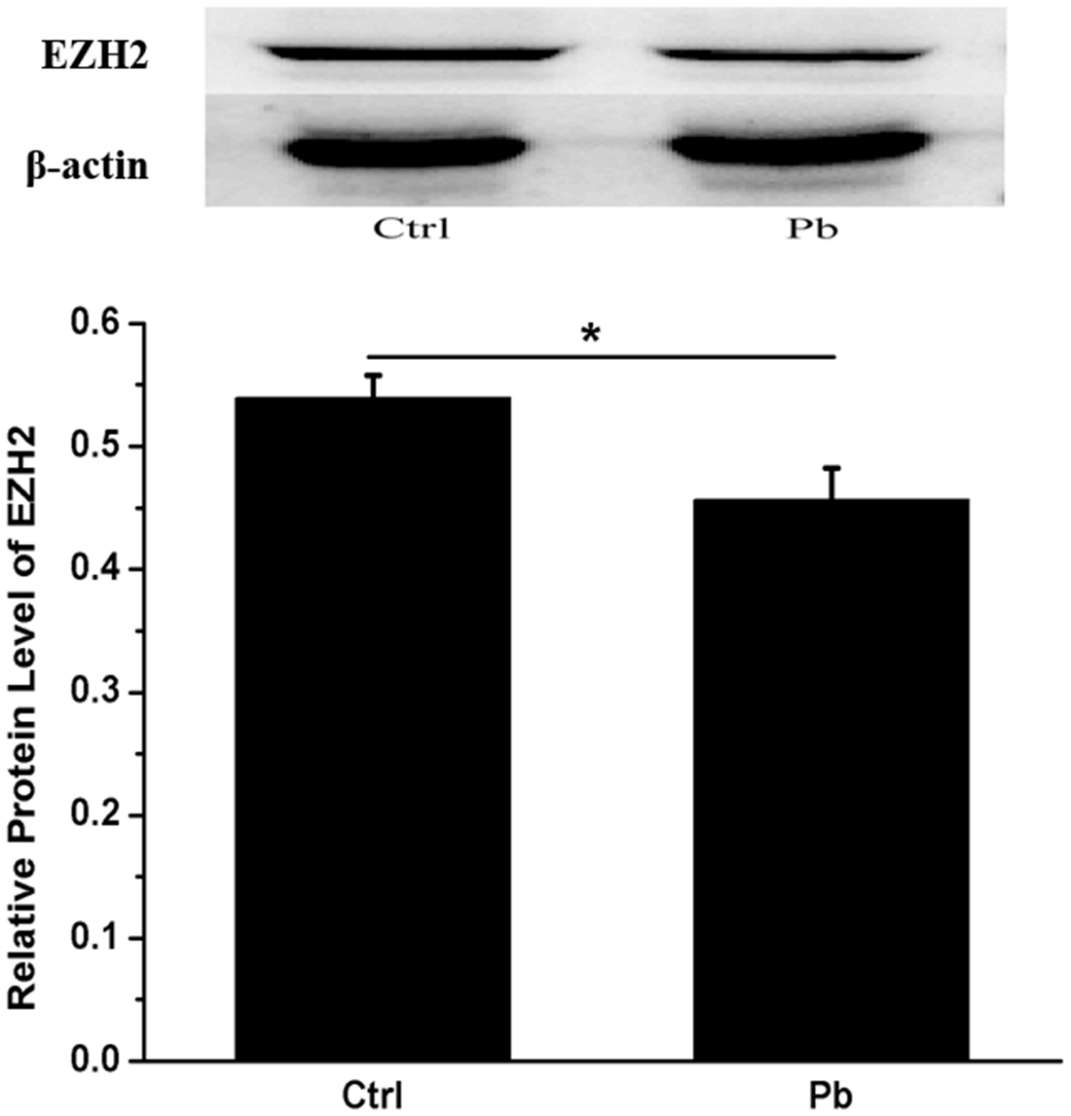
*In vivo* test of Pb’s effect on the expression of EZH2 in the rat hippocampus The rats were administered with 250 ppm’s lead acetate indirectly from their mothers and directly from weaning, and the control (receiving no Pb) and experimental rats were decapitated on PND 21, and their hippocampus were collected for Western analysis. Quantification of western blotting results for EZH2 was shown in the lower portion. The protein level of EZH2 was calculated as compared to that of beta-actin. Data were shown as mean ± SEM (n=6). * Indicated significantly different (*P*<0.05). Representative western blots of EZH2 and β-actin (internal control) proteins were shown in the upper portion.

In addition, the inhibitory effect on EZH2’s expression appeared to be enhanced by prolonged incubation time (Figure 3). Both of 8 h and 12 h’s treatment did not produce any significant differences between control and Pb-treated group. However, as the exposure period was extended to 24 h, EZH2’s expression was reduced to a considerably lower level (0.2520±0.01104 and 0.1610±0.02086 for control and Pb-treated group at 24 h, respectively). The findings here indicated that EZH2’s protein levels were influenced by Pb exposure in a temporally dependent manner. Besides, EZH2’s down-regulation induced by Pb’s administration was further validated *in vivo* in the rat hippocampus (Figure 4).

### EZH2 mediated Pb-induced neurotoxicity

**Figure 5:**
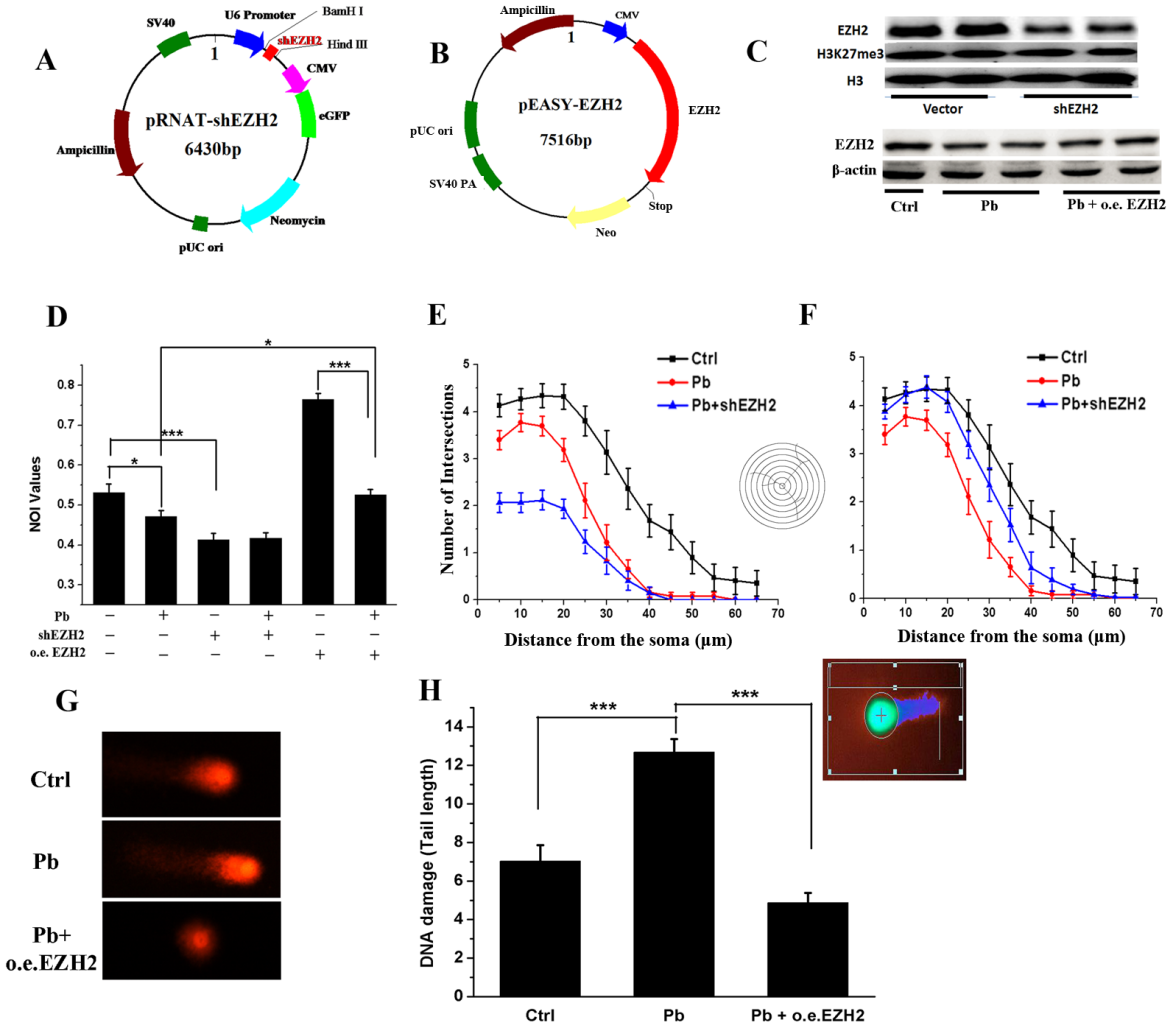
EZH2 mediated the lead-led impairment of neurite outgrowth of PC 12 cells **(A, B)** Schematic representation of pRNAT-shEZH2 and pEASY-EZH2 vector. **(C)** Western blotting sampled from EZH2-knocking down and overexpressing cells. EZH2, H3K27me3 antibodies were used to perform the trials, and H3 was used as the internal control. **(D)** NOI (Neurite Outgrowth Index) values of PC 12 cells in the presence and absence of Pb, shEZH2 and o.e. EZH2 treatment. NOI was calculated as the percentage of cells harboring at least one neurite with length equal to the cell body diameter of the total cells in the field. The cells were sampled from at least 15 visual fields for each group. **(E, F)** Sholl analysis of PC 12 cells transfected with pRNAT-shEZH2 (E) or pEASY-EZH2 (F) vector. At least 15 cells for each group are analyzed, and error bars indicate S.E.M. **(G, H)** Representative figures (G) and DNA damage statistics (H) of PC 12 cells transfected with pEASY-EZH2 in the Comet assay (*n* = 50). The insert is a representative cell analyzed by the software CASP.

**Figure 6:**
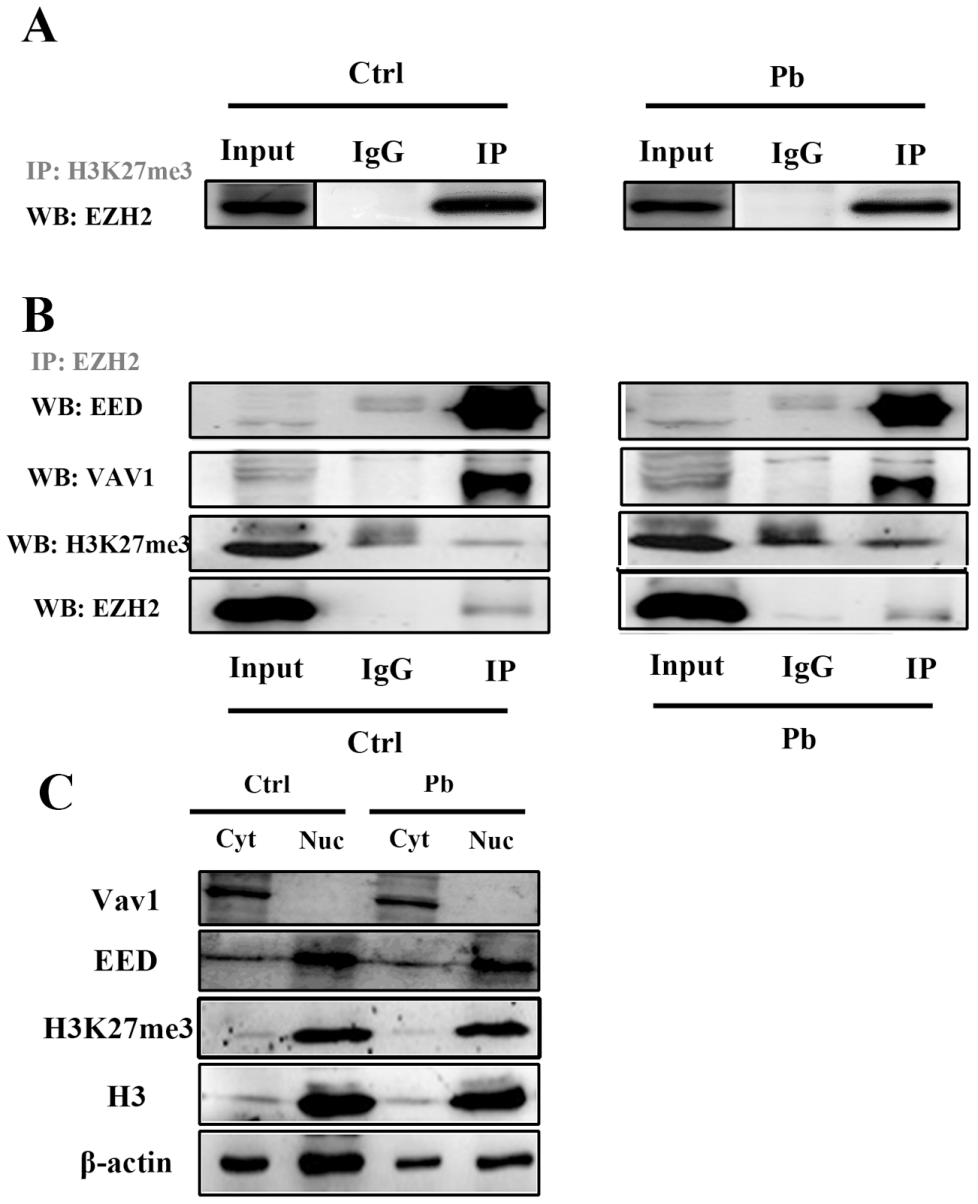
Co-immunoprecipitation (Co-IP) of H3K27me3 **(A)** and EZH2 **(B)** in Pb-treated and untreated PC 12 cells. IgG represents a control antibody used for IPs. Antibodies used for IP and Western blotting (WB) were labeled as grey and black, respectively. Prior to carrying out the IP experiments, one tenth of total lysates were subjected to the respective WB as input controls. **(C)** Expressions of Vav1, EED, H3K27me3 in the nucleus and cytosol were analyzed by western blotting. The nuclear (Nuc) and cytosolic (Cyt) cell extracts were collected from PC 12 cells with or without lead exposure (24 h), and subsequently quantified by WB with the corresponding antibodies. Expression of β-actin and H3 was used as the internal control for cytosolic and nuclear proteins, respectively.

In order to explore whether EZH2 directly mediated Pb-induced neurotoxicity, knocking down (KD) (Figure 5A) and overexpression (OE) constructs (Figure 5B) were established based on the backbones of pRNAT-U6.1-Neo and pEASY-blunt M2 plasmids, respectively. The constructed vectors were then transfected into undifferentiated PC 12 cells previously exposed by Pb, to examine the effect of *EZH2*’s interference on the impaired neurite outgrowth. As shown in Figure 5C, shEZH2-transfected cells exhibited a markedly reduced EZH2 level, ensuring the efficacy of the KD assays. Concomitantly, oeEZH2’s transfection largely offset the repressive effect of Pb on EZH2’s expression. Besides, H3K27me3 seemed rather resistant to any significant changes either from EZH2’s KD (Figure 5C) or Pb exposure (Figure 6C). EZH1’s compensating roles or low activity of demethylases might be responsible for its stability in this studied cellular context.

Subsequently, NOI (neurite outgrowth index) and sholl analysis were carried out to explicitly depict the neurite outgrowth profiles of PC 12 cells. According to Figure 5D, the percentage of cells with soma-sized neurites decreased significantly due to Pb treatment, which was then partially recovered through EZH2’s overexpression. Meanwhile, knocking down EZH2 yielded a similar impairment with Pb exposure. Along with the results derived from sholl analysis (Figure 5E and 5F), both loss- and gain-of-function assays markedly changed the intricate neural responses towards Pb treatment. And in a Comet assay (Figure 5G and 5H), EZH2’s overexpression dramatically rescued the cell damage caused by Pb exposure (10 μM). These are direct proofs that Pb brings in its neurotoxicity through the disturbance of normal activities of EZH2.

### EZH2 acted in a polycomb-dependent and independent way

EZH2 is primarily proposed as the catalytic subunit of PRC2, a protein complex also consisting of EED, Suz12 and RbAp48. The leading cellular function of this complex is to induce the trimethylation of the histone H3 lysine 27 (H3K27me3) [11], which is considered to be a crucial repressive mark to repress a quantity of genes’ expression. To investigate whether the decreased EZH2 levels dampened its recruitment to PRC2 and consequently led to a weakened histone modification, the co-immunoprecipitation assays were carried out in Pb-exposed PC 12 cells.

According to Figure 6A, EZH2 still exhibited a strong affinity with H3K27me3, but the levels of immnoprecipitated EZH2 by anti-H3K27me3 antibody were decreased in Pb-treated group. It seems that the H3K27me3’s position was less occupied by EZH2, suggesting that EZH2’s roles as causative agent in epigenetic regulation were diminished by Pb exposure. Considering the functional distribution, it was shown that a decreased fraction of EZH2 maintained its interaction with EED (Figure 6B), or, was recruited to the PRC2 complex. Strikingly, the hampered PRC2 assembly did not lead to a direct effect on EZH2’s association with H3K27me3 (Figure 6B), a finding suggestive of EZH2’s presence in a PRC2-independent fashion.

EZH2 was previously documented to play roles in the extra-nuclear signaling processes via the interaction with Vav1, which is a GTP/GDP exchange factor and linked with actin polymerization, in the T cells or fibroblasts [20], it was thus demonstrated that the similar form of interaction also existed in the neural cells (Figure 6B). In light of the findings here, EZH2 apparently distributed a substantial portion of function into its interplay with Vav1, justifiably linked with the cytoskeleton’s extension independent of EZH2’s epigenetic roles. Of note, Pb exposure also caused a marked decline of the EZH2-Vav1 interaction.

Subsequently, we also investigated the cellular location of EZH2’s functional partners. Based on Figure 6C, EED and H3K27me3 were predominantly present in the nuclei, while Vav1 maintained its presence almost exclusively in the cytosol. That is an indication that EZH2’s sub-functionalization was divergent, that is, PRC2 assembly and Vav1’s signaling did not exhibit any functional redundancy. It was also revealed from Figure 6C that, the expression of VaV1, EED or H3K27me3 were not significantly altered in the context of Pb exposure, indicating that the diminished EZH2-EED or EZH2-Vav1 levels should not be attributed to the impaired availability of functional partners. Collectively, Pb exposure exerted deleterious effects on the multiple signaling or regulatory processes EZH2 engaged.

### EZH2’s compartmentalization was remodeled upon Pb exposure

**Figure 7:**
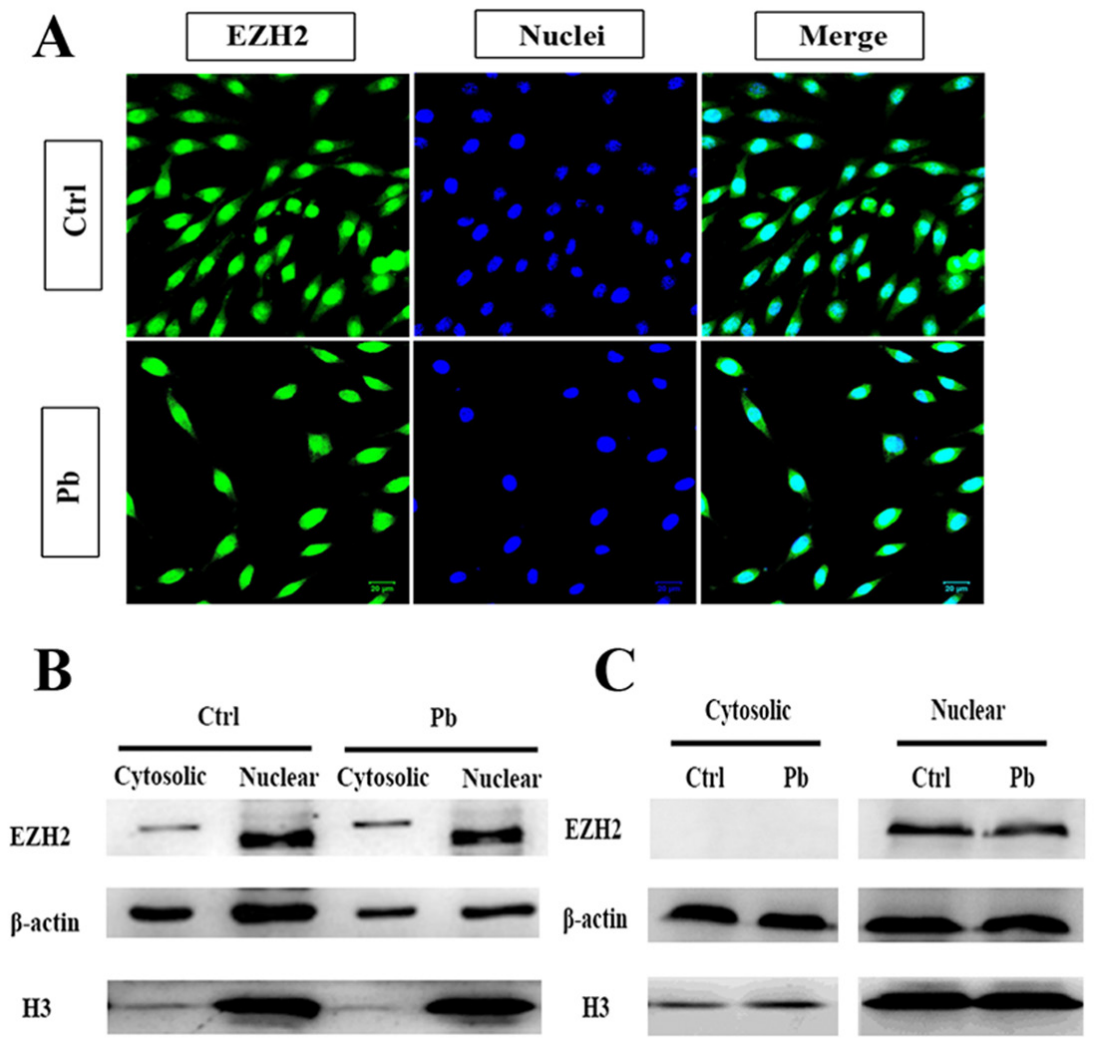
Cytosolic and nuclear compartmentalization of EZH2 in the context of lead exposure **(A)** IF staining of EZH2 on the coverslip of PC 12 cells with or without lead exposure. Nuclei was stained with DAPI, and bars represent 20 μM. **(B, C)** Expression of EZH2 in the nucleus and cytosol was analyzed by western blotting. The nuclear and cytosolic cell extracts were collected following 24 h (B) or 36 h’s (C) exposure, and subsequently quantified by WB with anti-EZH2 antibody. Expression of β-actin and H3 was used as the internal control for cytosolic and nuclear proteins, respectively.

Given the alterations of sub-functional distribution, further steps were taken to explore whether there was any aberrant state of EHZ2 for itself. Its cellular localization was then investigated through IF (Immunofluorescence, Figure 7A) and WB (Figure 7B and 7C) assays. The immunofluorescent figures unambiguously validated the cytosolic and nuclear presence of EZH2, with nuclear fractions overwhelmingly predominant. Pb treatment was found to severely influence the total amount of EZH2 (Figure 7A). Specifically, as shown in WB, the EZH2’s alterations with respect to cytosolic and nuclear fractions did not act in concert: treated with Pb, the cytosolic EZH2 showed an increment to some degree at 24 h and markedly reversed to disappearance at 36 h, whereas the nuclear fraction only showed a stepwise attenuation as exposure prolonged (Figure 7B and 7C). It suggested an interesting mode of EZH2’s redistribution: in the first 24 h, Pb treatment removed a fraction of EZH’s nuclear activity and reinforced its presence in cytosol; subsequently, as exposure lasted to 36 h, the cytosolic EZH2 rapidly disappeared, with Pb’s effect exclusively manifested as the changes of nuclear EZH2. On the basis of these observations, EZH’s compartmentalization in PC 12 cells appeared to be remodeled upon Pb exposure.

### EZH2’s occupancy on multiple genes were altered by Pb exposure

As a transcriptional repressor, EZH2 was regarded to play its roles primarily through the inhibition of target genes’ expression. Relying on the data derived from ChIP-on-chip experiments that we previously performed using H3K27me3 antibody on Pb’s toxicity (unpublished data), as well as their nerve-related activities, six genes, namely *Alox15*, *Notch1*, *NGFR*, *EGR2*, *HFE* and *CaMKK2*, were chosen as competent candidates to be checked for their dynamics regulated by EZH2.

**Figure 8:**
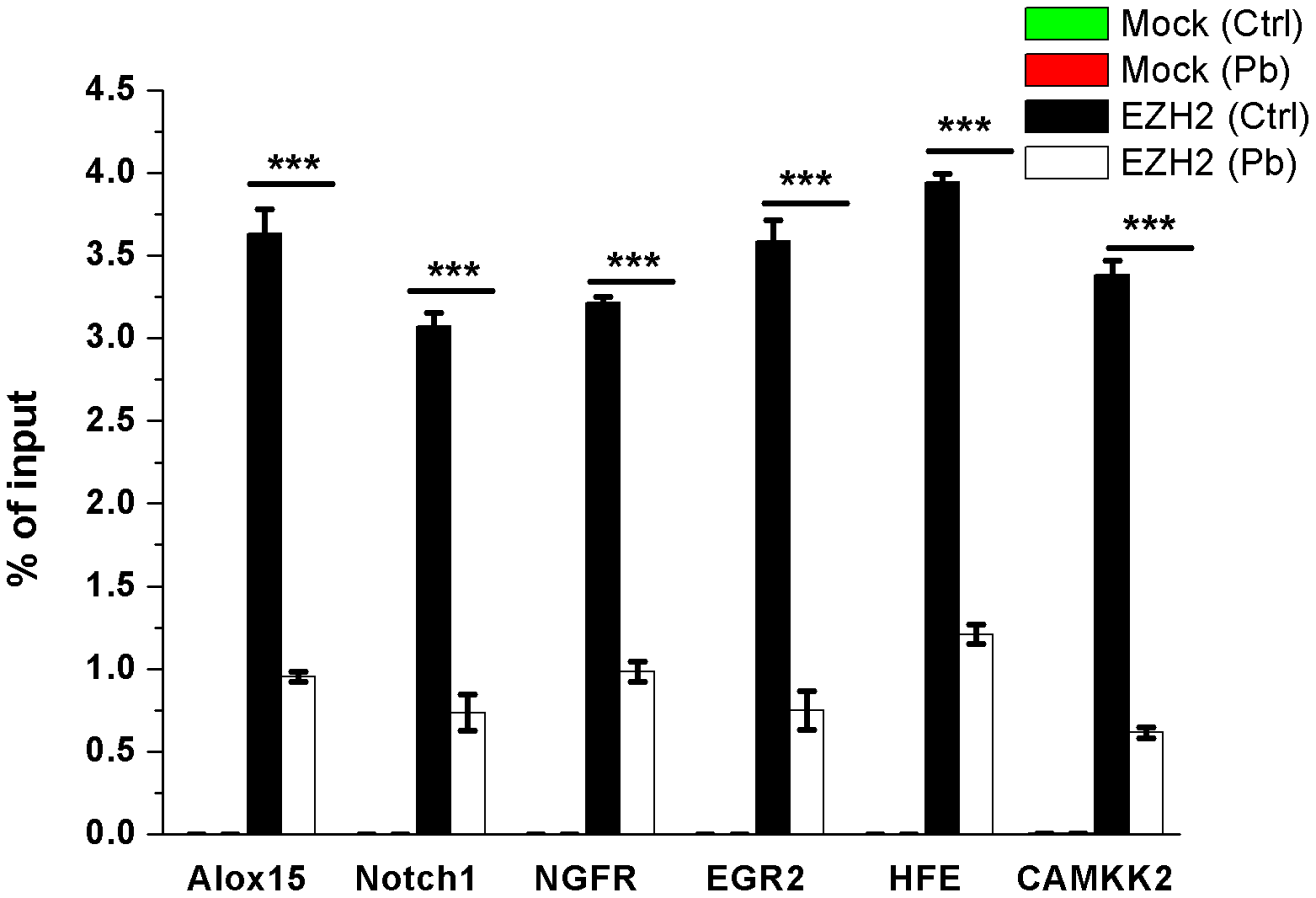
ChIP analysis of *Alox15*, *Notch1*, *NGFR*, *EGR2*, *HFE*, *CaMKK2* promoter regions occupied by EZH2 in both control and Pb-exposed cells Antibodies of IgG (mock) and EZH2 were used to perform the ChIP assay, respectively. Percentages of input DNA are shown as the means ± SEM of triplicate independent experiments. *** Indicates the statistical significance of differences of *P*<0.001.

A chromatin immunoprecipitation (ChIP) assay was conducted to investigate the enrichment profiles of EZH2 on the candidate genes’ promoters. As shown in Figure 8, EZH2’s recruitment on all of the tested genes was significantly reduced in response to Pb exposure. This *in situ* inspection provided evidence that EZH2 posed its inhibitory effect through its interaction with the objective genes, and this regulatory potency was dampened by the Pb-induced deficiency of EZH2.

### EZH2 mediated diverse molecular aspects concerning Pb neurotoxicity

**Figure 9:**
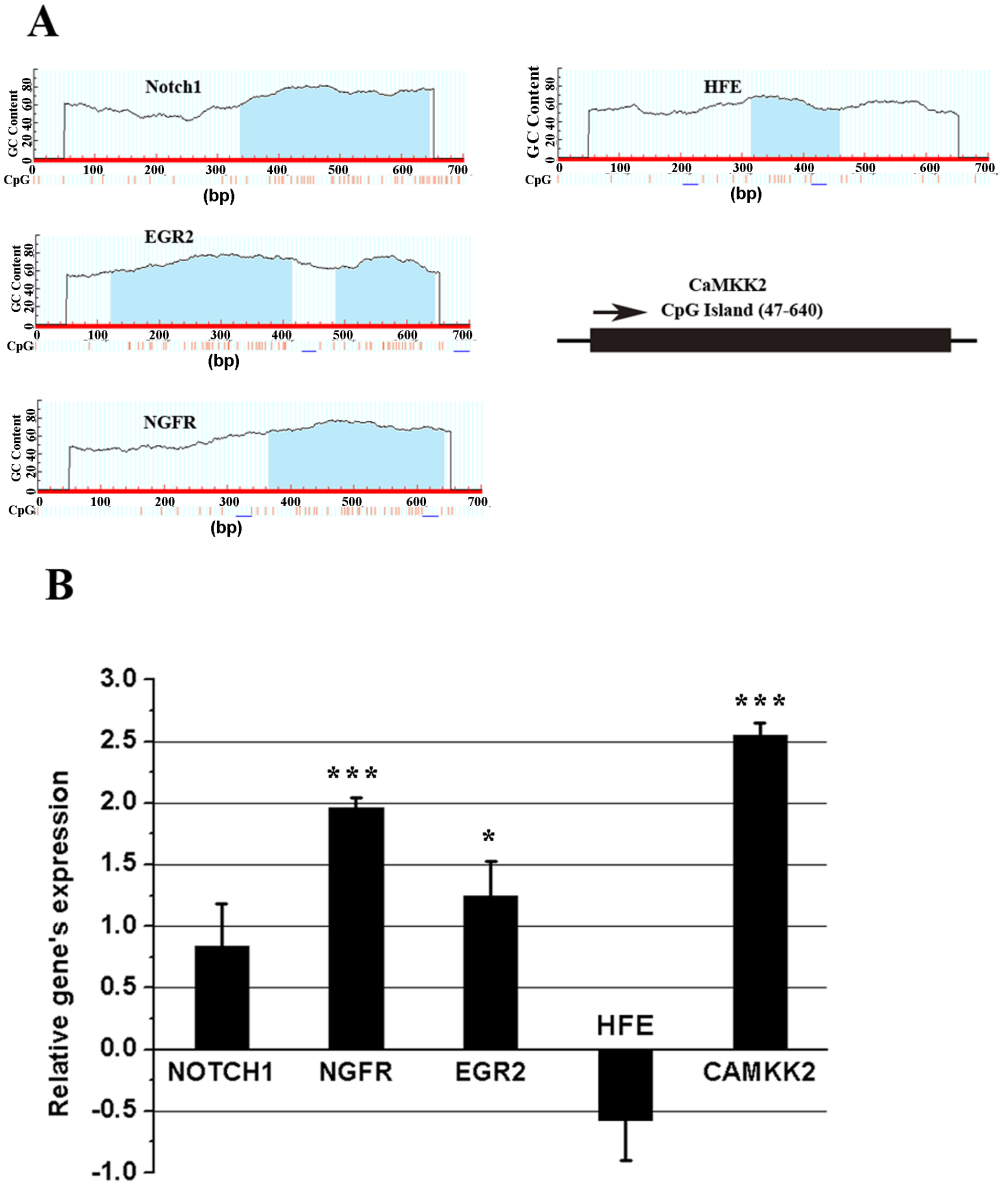
Promoter CpG islands’ prediction **(A)** and qRT-PCR’s analysis of mRNA levels **(B)** of *Notch1*, *NGFR*, *EGR2*, *HFE*, *CAMKK2* in response to Pb treatment. (A) “Methprimer” was used to perform the CpG island prediction of each gene’s promoter region, termed as the “-500 ∼ +200” region of TSS (Transcription Start Site). The predicted CpG islands (CGI) were marked as the blue shadow. The schematic representation was shown for *CAMKK2*’s CGI profiles to comply with the calculated result, due that the prediction picture could not be properly exhibited by Methprimer software, in this instance; (B) the fold change indicates the relative change in expression levels between the presence and absence of Pb’s treatment on PC 12 cells. The expression value of each control sample was normalized to “0”, and fold changes are expressed as the relative alterations of Ct values. Fold changes are shown as the means ± SEM of triplicate independent experiments. *** and * indicate the statistical significance of differences of *P*<0.001 and *P*<0.05, respectively.

To verify that the functional genes’ expression was genuinely stimulated through EZH2’s suppression, their transcriptional levels were determined using fluorescence quantitative real-time PCR. As evidenced by Figure 9B, upon Pb treatment, the expression of *NGFR*, *EGR2* and *CaMKK2* was considerably up-regulated, a consequence accordant with EZH2’s negative regulation. Conversely, both *Notch1* and *HFE*’s expression did not show any significant differences between control and Pb-treated cells. The findings here might suggest that EZH2 played dominant roles in governing the Pb-induced molecular changes of *NGFR*, *EGR2* and *CaMKK2*, while additional *cis*- or *trans*- elements complicated the regulation of the other two genes.

To test this possibility, the CpG Islands within the promoter region of the respective gene were identified and analyzed using Methprimer. It was seen from Figure 9A that, all of the tested genes harbored at least one entire CpG island, consistent with the argument that PRC2 is preferentially recruited to a CpG-rich region [21]. Otherwise, *HFE* seems to harbor a comparatively small size of CpG island (less than 200 bp) (Figure 9A), implicating that the intrinsic CpG methylation likely prevented the recovery of *HFE*’s transcription following the removal of EZH2’s repressive activity.

**Figure 10:**
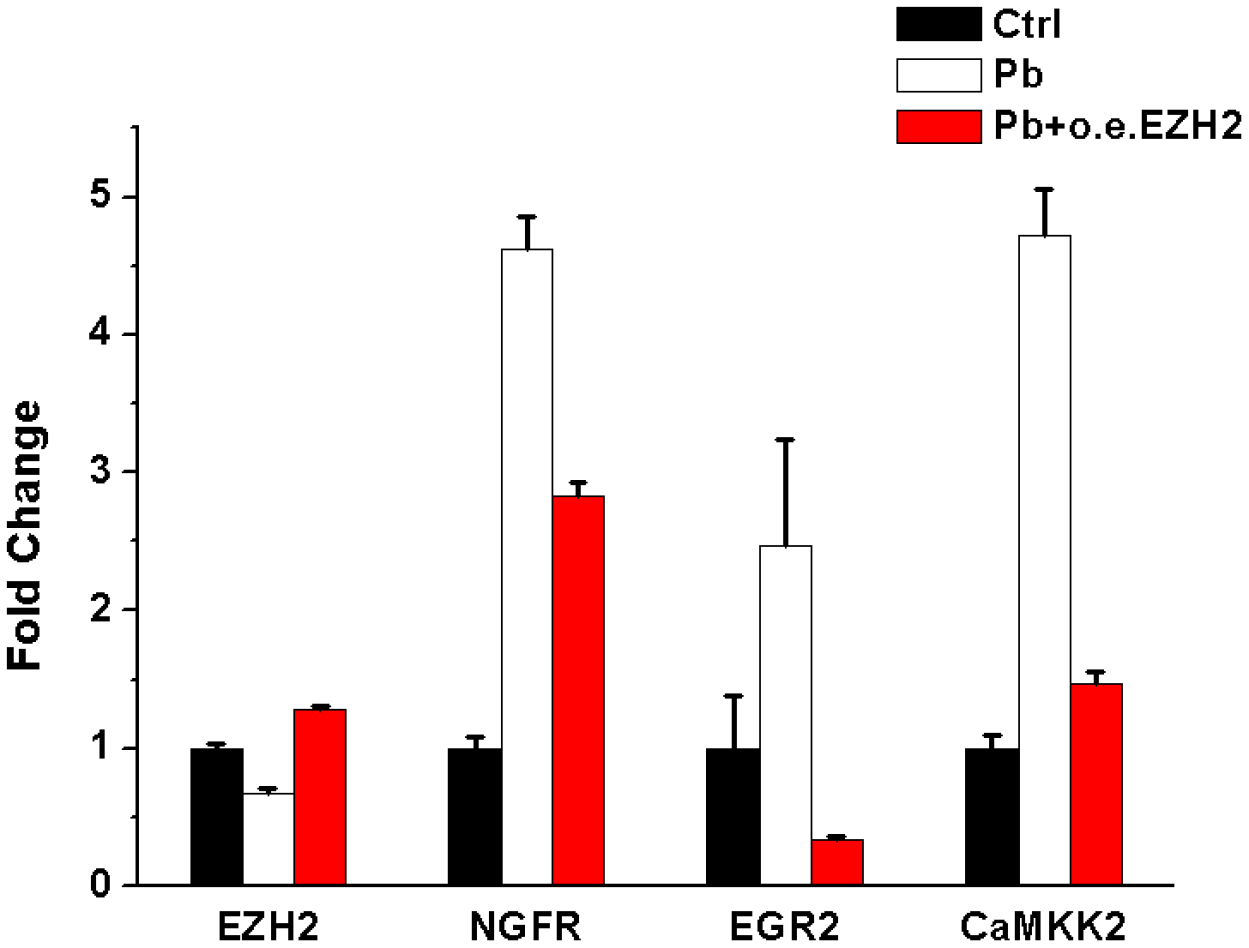
qRT-PCR’s analysis of mRNA levels of *EZH2*, *NGFR*, *EGR2*, *CAMKK2* upon EZH2’ overexpression following Pb’s treatment The expression value of each control sample was normalized to “1”, and fold changes are given as percentages of control after normalization. Fold changes are shown as the means ± SEM of triplicate independent experiments. The expression levels of each gene showed significant difference (*P*<0.05) in the comparisons of groups pertaining to control-Pb and Pb-oeEZH.

We next investigated whether the target genes’ expression levels could return to normal following EZH2’s rescue. According to Figure 10, with *EZH2*’s expression recovered, the mRNA levels of *NGFR*, *EGR2*, *CaMKK2* were significantly reversed to a variable extent. Combined with the striking phenomenon derived from neurite outgrowth (Figure 5), it’s suggested that in the course of Pb intoxication, EZH2 directly mediated the dynamic changes of several crucial functional molecules and the resulting phenotypic impairments.

## DISCUSSION

Mounting evidence has proposed that low-level Pb exposure is able to elicit a cascade of adverse neurotoxic consequences [22, 23], but the exact molecular aspects underlying this noted toxicological process remains to be elucidated. Sanders, Liu [5] summarized the relevant studies and proposed oxidative stress, membrane bio-physics alterations, deregulation of cell signaling, and impairment of neurotransmission as key mechanisms involved in Pb neurotoxicology. Interestingly, it was found that (Figure 9) some key molecules, namely EGR2, NGFR and CaMKK2, representative of their respective pathways were simultaneously dysregulated as Pb exposed. Specifically, EGR2 is an early-acting transcriptional factor responsible for a range of signaling pathways [24], CaMKK2 is involved in the intracellular calcium homeostasis and neuronal transmission [25], and NGFR contributes to the axonal growth and nerve damage repair [26]. It was thus tempting to speculate that Pb exerted its detrimental effect on the neural cells through a complexity of molecular network, specifying neural development, transmission and repair mechanisms. With key genes’ involvement, Pb-led neurotoxicity is multi-targeted and manifested pervasively.

EZH2 was supposed to exert its transcriptionally inhibitory effect mainly through H3K27me3 mark it catalyzed. It’s very intriguing, however, to unravel that EZH2 and H3K27me3 acted divergently in this context, as demonstrated both in Co-IP (Figure 5) or KD (Figure 6) assays. The similar observations were also previously documented [27, 28], wherein the authors explained that a homolog of EZH2, EZH1, probably compensated for EZH2’s loss whenever is needed. This possibility could not be ruled out when EHZ2 is insufficient by Pb treatment or artificial interference, but it’s argued [29] that EZH1 could only transiently interact with other PRC2 components, preventing it playing essential roles in H3K27me3’s alterations. It deserves to be noticed that H3K27me3 is a comparatively stable mark, suggesting there existed a dynamic mechanism to maintain its proper level. This mechanism might be characterized by EZH1’s complementary roles, or by the deficiency of potent demethylases, or both. Actually, the transcription levels of *JMJD3* and *UTX*, two major H3K27me3 demethylases, remained relatively low and did not show any changes with Pb exposure (unpublished data). Thus H3K27me3’s stable levels upon Pb exposure might be attributable to a sustained consequence of the preceded EZH2’s activity. Regardless of the precise mechanisms, it’s here demonstrated that in the cellular and toxicological context involved in this investigation, EZH2 is more prone to dynamic and diversified changes than its epigenetic counterpart.

EZH2 is a multi-functional molecule, despite that it’s often solely regarded as a catalyzing component of PRC2 complex. EZH2 directly interacts with various transcription factors [30], or it inhibits some genes’ expression as a co-repressor *in trans* [17]. EZH2 even serves to release its activity independent of transcriptional regulation [31]. The proposition that EZH2 acted in the presence and absence of PRC2 assembly was also validated in the neurocellular models exposed by Pb (Figure 6), with PRC2’s involvement still occupying the predominant positions. In the cytosol, EZH2 was directly associated with Vav1, a similar phenomenon with that in the antigen receptor signaling of T cells [20]. On the basis of these findings, EZH2’s multiple roles, specifically PRC2-coupled and uncoupled, were implicated in this neurotoxic process. Overall, EZH2’s abundance was reduced, probably leading to the weakened roles in every signaling pathway it governs. However, concomitant with this tendency, a relatively larger fraction of the remaining EZH2 was still transferred into the chromatin, to ensure its interaction with H3K27me3 (Figure 6). The H3K27me3 preference might be due to its central roles in maintaining the fundamental cell identity, as suggested previously [32].

In the process of these Pb-induced changes, a couple of interesting phenomenon was observed: (1) at 24 h’s exposure, EZH2’s interaction with Vav1 was reduced while the total cytosolic quantity of EZH2 was augmented; (2) EZH’s compartmentalization with respect to cytosol and nuclei was changed markedly when Pb exposure was prolonged to 36 h. The first discovery explicitly indicated that except from Vav1, EZH2 still possessed other cytosolic partners to comply with other signaling. This argument could be supported by the findings that EZH2 binds Wnt effector β-catenin and promotes transcriptional activation of genes in breast cancer cells [27]. The peculiarity of the second discovery lies that EZH2’s cytosolic fraction was temporarily enhanced and rapidly declined to disappearance. This is an indication that the unidentified cytosolic roles of EZH2 simply persisted transiently. Further, it seems that the changing profiles of EZH2, upon Pb treatment, were strictly exposure timing-dependent. Collectively, a proposition could be raised: classified by Pb exposure, distinct EZH2’s functional landscape was presented through a mixed action of time, location and functional chaperones.

**Figure 11:**
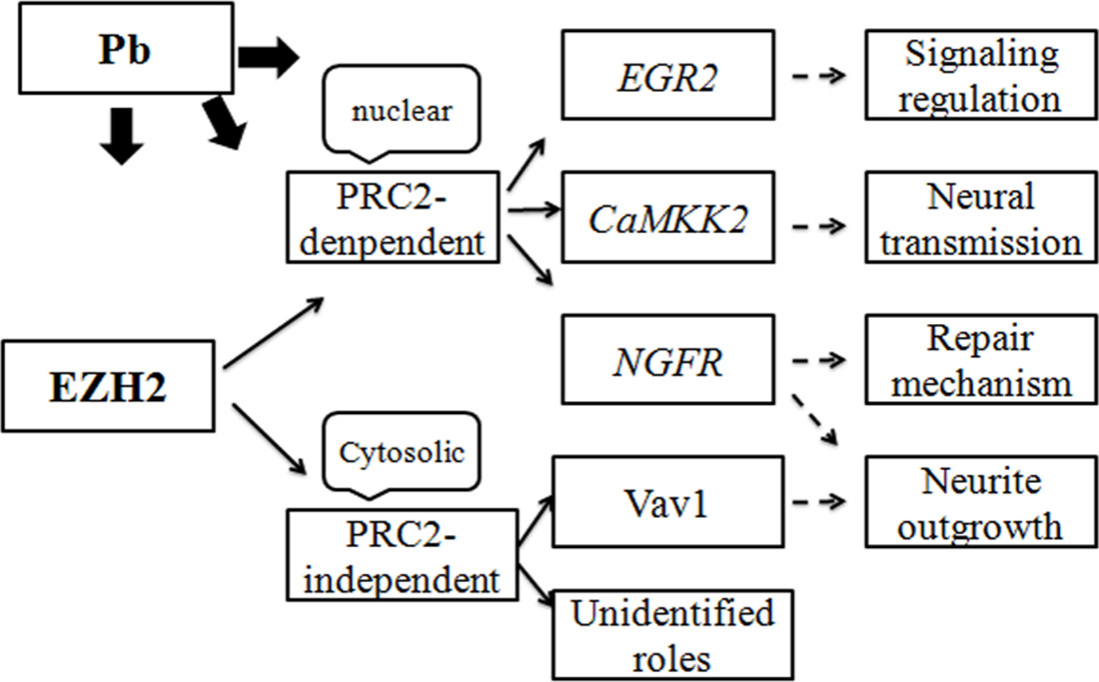
Schematic representation of EZH2’s roles in lead-led neurotoxicity The proteins (or genes) and the neurological processes involved in Pb-lead neurotoxicity were indicated with hollow boxes. The subcellular location and mode of action of EZH2 was also shown in the same way. The arrows indicate the regulatory or functional relationships between the individual elements.

In light of its leading status as transcriptional repressors, EZH2’s regulatory activity was mainly achieved through fine-tuning the expression of its downstream genes. It’s discovered in this study that, mediated by EZH2, Pb treatment triggered a range of nerve-functional genes’ abnormal reaction (Figures 8 and 9). Representative of various neurotoxic aspects, such as neural development, transmission and repair mechanism, these genes’ expression continued to be reprogrammed by EZH2’s attenuation and artificial rescue. Consequently, bridged by its target molecules, EZH2’s activity were mechanistically linked with diverse mechanisms underlying Pb intoxication. Along with its transcriptional-independent roles, EZH2 was supposedly centered at the regulation of neural impairment and stress response induced by Pb. Therefore, based on the results presented here, a route map concerning EZH2’s pivotal roles in Pb-led neurotoxicity was proposed (Figure 11), favoring the better illustration of this environmental etiology of neurological impairments from the molecular perspective.

In summary, in addition to cancer biology, EZH2 was, for the first time, proposed as a central player in Pb-induced neurotoxicity in this study. With detailed spatiotemporal changes investigated, EZH2 was shown to exert its regulatory effect through remodeling its sub-functionalizaion, as well as transcriptionally reprogramming its downstream genes. This is an interesting example to suggest a common molecular signature pertaining to Pb-led neurotoxicity, and provided a promising molecular target for medical intervention. Of note, as shown in the gain-of-function trials, EZH2’s overexpression did partially rescue the dampened outgrowth profiles of neurites, an excellent proof that EZH2’s interference could be used to counteract the harmful outcomes brought by Pb poisoning, and future attempts are still warranted to validate its consistent efficacy *in vivo*.

## MATERIALS AND METHODS

### Reagents and antibodies

The following antibodies were used in this study: anti-EZH2 (Cell Signaling, Beverly, MA, USA), anti-H3K27me3 (Millipore, MA, USA), anti-EED, anti-Vav1 (Proteintech, Wuhan, China). Acrylamide, bis-acrylamide, β-mercaptoethanol, Hybond nitrocellulose, sodium dodecyl sulfate, Tris, and lead acetate (with the final concentration fixed to 1, 2.5, 5, 10, 20 μM, respectively) were purchased from Sinopharm Group (Beijing, China). All other reagents were of the highest analytical grade.

### Cell culture

PC 12 cells (undifferentiated and differentiated lines, CAS, Shanghai, China) were cultured at 37°C, 5 % CO_2_ in humidified atmosphere with RPMI1640 medium supplemented with 5 % FBS (Fetal Bovine Serum), 10 % HS (Horse Serum) and 1 % penicillin-streptomycin. Cells were re-inoculated at least twice before being subjected to the Pb treatment. Cells were plated onto 6-well tissue culture plates coated with poly-D-lysine/laminin. Lead acetate with various final concentrations (1, 5, 10, 20 μM) was added when cells were grown for 24 h, as 10 μM Pb’s exposure was generally used in low-level exposure studies in PC 12 cells [33]. Subsequently, cells were allowed to continue its growth for 24 h or 36 h. For undifferentiated cell lines, 24 h after plating, the medium was replaced with fresh medium containing 0.5 % FBS, 1 % HS and 1 % penicillin-streptomycin with NGF (50 ng/ml) with or without Pb exposure. Plasmid transfection was carried out as cells reach 50∼60 % and 40 % confluent for differentiated and undifferentiated cells, respectively.

### Animal test

Sprague–Dawley (SD) rats were obtained from the Laboratory Animal Center, Anhui Medical University, P.R. China. The pups (n=6 for each group) acquired Pb indirectly through their mothers, whose drinking water contained lead acetate (250 ppm), and then directly from weaning. The new-born rats (with or without Pb exposure) were allowed to grow to postnatal day 21 (PND 21) before they were decapitated and the hippocampus were collected and subjected to the subsequent protein quantitative analysis. All experimental operations comply with the National Institute of Health Guide for the Care and Use of Laboratory Animals and were approved by institutional animal care and use committee of Hefei University of Technology, China.

### Quantification of neurite outgrowth

Quantification of neurite outgrowth was carried out as described by Hashimoto et al. [34] with some modifications: 3 days following incubation (with medium changed every 36 h) with NGF (50 ng/ml), morphological analysis was performed according to the digitized images derived from fluorescence microscopy (Nikon, Tokyo, Japan). Differentiated cells were counted by visual examination of the field. The NOI values were expressed as the percentage of cells harboring at least one neurite with length equal to the cell body diameter of the total cells in the field. The Sholl analysis was performed using Image J software (NIH, USA), according to the developers’ instructions.

### Single cell gel electrophoresis assay (comet assay)

The Comet assay was carried out using the CometAssay Reagent Kit for Single Cell Gel Electrophoresis Assay (Trevigen, Maryland, USA), according to the manufacturer’s instructions. Briefly, following 48h’s incubation with Pb (10 μM) and/or transfected pEASY-EZH2 plasmid, the differentiated PC 12 cells were collected at a density of 10^5^ cells/ml. The cells were combined with molten LMAgarose at a ratio of 1:10 (v/v) at 37°C, and 50 μl was pipetted onto CometSlide. Immerse slides in Lysis Solution for 60 min at 4°C and in Alkaline Solution for 20 min at room temperature for unwinding the DNA helix. The slides were subjected to gel electrophoresis at 21 volts for 30 min. Following the staining with SYBR Gold, the digitized images were derived from fluorescent microscopy (Nikon, Tokyo, Japan). The DNA damages were analyzed using the software CASP, and tail length was used to indicate the DNA damage and repair index. At least 50 comets were scored for each treatment and the experiment was repeated three times.

### Plasmid construction

The vector pRNAT-U6.1-Neo (Genescript, Beijing, China) was used to construct the EZH2-shRNA-expression vector. The annealed shRNA fragment was ligated into the *BamH* I/*Hind* III restriction sites of the vector, resulting in the pRNAT-shEZH2. The target sequence of shEZH2-1 and shEZH2-2 were 5’-GCTGACGAAGTAAAGACTATG-3’ and 5’- GCTGACGAAGTAAAGACTATG-3’, respectively. As shEZH2-2 was proved to be more efficacious in inhibiting EZH2’s protein levels, it was then utilized in the subsequent assays. A vector targeting the scrambled sequence was also established as a negative control.

The vector pEASY-blunt M2 (TransGen, Beijing, China) was used to construct the EZH2-overexpression vector. The ORF of rat EZH2 was amplified and ligated to the blunt ends downstream of CMV promoter, resulting in the pEASY-EZH2. The resultant constructs were subjected to nucleotide sequencing to ensure the fidelity of genetic manipulation. A vector with a scrambled sequence was also established as a negative control. All transfections were carried out using Lipofectamine 2000 (Thermo, Shanghai, China) with 3 μg and 1 μg total plasmids for differentiated and undifferentiated PC 12 cells, respectively.

### MTT reduction assay

Cell viability was assessed by the MTT reduction assay as described previously [9] with some modifications: PC 12 cells were seeded into 96-well plates at a concentration of 2×10^4^ cells/well. 12 h later, the medium containing varying concentrations of Pb was added to cells and incubated for 24 h. 50 μL of MTT-PBS solution (5 mg/ml) was added to the culture and incubated in the dark for 4 h at 37°C. The supernatants were aspirated and the formazan crystals in each well were dissolved in 50 μL of DMSO. The relative amount of the MTT reduction was determined based on the absorbance at 570 nm using a plate reader. The cell viability of the untreated group was then normalized as 100 %.

### Western blot analysis

PC12 cells were washed with PBS and lysed in Laemmli lysis buffer. The cytosolic and nuclear fractions were separated using ProteinExt Mammalian Nuclear and Cytoplasmic Protein Extraction Kit (TransGen, Beijing, China), according to the manufacturer’s instruction. The total proteins of each sample were quantified using BCA protein assay kit (Beyotime, Shanghai, China). Equal aliquot (25 μg) of the proteins were loaded onto a 12 % SDS-PAGE for electrophoresis. The separated proteins were then transferred to PDVF membrane (Millipore, MA, USA). For immunodetection, the blots were blocked for 1 h in TBST (50 mM Tris/HCl, pH 7.8, 0.13 M NaCl, 0.1% Tween 20) containing 5 % nonfat dry milk at room temperature, followed by incubation with the primary antibodies overnight at 4°C. After incubation with the second antibody and extensive washing, immunoreactivity was detected by ECL plus Western Blotting Detection system (Thermo, MA, USA). The band intensity was normalized to the internal control (β-actin for whole and cytosolic extracts, H3 for nuclear extracts) for comparisons.

### Immunofluorescence

24 h later following Pb’s administration, immunofluorescence was performed according to the method of Stansfield et al. [6]. Briefly, PC 12 cells grown on coverslips were rinsed in PBS and fixed in 4 % paraformaldehyde, followed by another fixation in ice-cold methanol. 0.2 % of Triton X-100 was added to permeabilize the membranes and subsequently 10 % of normal goat serum was added to block the process. Fixed cells were incubated in anti-EZH2 antibody overnight at 4°C with the dilution of 1:1000. Subsequently, coverslips were incubated in the second antibody diluted in block solution. Coverslips were mounted onto slides in Prolong Gold mounting media with DAPI (Invitrogen, Shanghai, China). Immunofluorescent-labeled cells were imaged at 40× magnification using fluorescence microscopy (Nikon, Tokyo, Japan).

### Quantitative RT-PCR analysis

The transcriptional levels of the objective genes were measured through quantitative real-time RT-PCR protocols. First, the total RNAs were extracted from PC 12 samples using the PureLink RNA mini kit (Thermo, Shanghai, China). Subsequently, the reverse transcription reaction was completed according to the manufacturer’s instruction (TransGen, Beijing, China), resulting in the first strand of total cDNA.

Real-time PCR was performed on cDNA using the Roche LightCycler 96 (Shanghai, China). The 20 μl reaction pool of qPCR was composed of: 10 μl of SYBR premix Extaq; 0.6 μl of forward and reverse primer each; 1 μl of cDNA template (10 times dilution) and 7.8 μl of deionized water. The primers used in this study were listed in Table 1. The transcriptional levels were first calculated as the -∆Ct (threshold cycle) value’s difference from that of 18s rRNA under the same conditions, and normalized with -∆Ct (untreated group set as “0”) or 2^-∆∆Ct^ (untreated group set as “1”) for Pb-treated and gene disturbance assays, respectively.

**Table 1:** Primers used in this study

Primers	Sequences (5’-3’)	Methods
CAMKK2F	ATCCCTGTCCTACTCACCA	q-RTPCR
CAMKK2R	TTTCATCCTTCAGCGTGT	q-RTPCR
NGFRF	ACCTCATTCCTGTCTATTGCT	q-RTPCR
NGFRR	CGCCTTGTTTATTTTGTTTG	q-RTPCR
EGR2F	ACCACCTCACCACTCACA	q-RTPCR
EGR2R	ACTGCTCTTCCTCTCCTTCT	q-RTPCR
HFEF	CCCCTGGAGAAGAGACAA	q-RTPCR
HFER	AACAAAGAAGATGGCACAAA	q-RTPCR
Notch1F	CGCCCTTGCTCTGCCTAAC	q-RTPCR
Notch1R	CACTTCGCACCTCCCTCCA	q-RTPCR
18SrDNAF	TGAGTCCACTTTAAATCCTTTAACGA	q-RTPCR
18SrDNAR	CGCTATTGGAGCTGGAATTACC	q-RTPCR
Alox15PF	ATTTGAATTTTTTATTGGGACACAC	ChIP
Alox15PR	TGCTAACAGCCATCACAACTACAT	ChIP
Notch1PF	TATTTCTGTAGCATTGGGCGAT	ChIP
Notch1PR	TTGGCACAAACAACACCTTCC	ChIP
NGFRPF	ATGTATGTCTTAGGAACCCTTGTAG	ChIP
NGFRPR	ATACACAAACACACTCTCAAGCAC	ChIP
EGR2PF	AGCGACGTCACGGGTTATTG	ChIP
EGR2PR	GAGCCAGGAGTTGCTGATGTAG	ChIP
HFEPF	GCCAAGCAAATACAAACCATC	ChIP
HFEPR	GTTAGCAACAGACGCATACCC	ChIP
CAMKK2PF	CCGTTCGGAGTGAGTGATG	ChIP
CAMKK2PR	GCAAGTTTGGGAGTGGGTC	ChIP

### Coimmunoprecipitation

Co-IP assay was performed according to Sen [35] with some modifications: Cells were collected and lysed in lysis buffer (50 mM Tris (pH 7.4), 150 mM NaCl, 0.1 % CHAPS, 100 μM deferoxamine, and 1 mM EDTA), incubated on ice for 15 min and cleared by centrifugation at 14000 g at 4°C for 20 min. The supernatant was transferred to a new tube, with a fraction pipetted out as input. IP buffer (50 mM Tris (pH 7.4), 150 mM NaCl, 0.1 % CHAPS, 100 μM deferoxamine, 1 mM EDTA, and 0.1 mg/mL BSA) was added to bring samples to a total volume of 500 μL. 5 μg of Anti-H3K27me3, anti-EZH2 antibody or unimmune IgG was added to the sample and incubated overnight at 4°C. Subsequently, protein A/G beads (1:100) were added and incubated on a rotator at 4 °C for 4 h. The beads were washed four times with lysis buffer and quenched with 30 μL SDS sample buffer. Coimmunoprecipitates were resolved by SDS-PAGE and analyzed by Western blotting with anti-EZH2, anti-Vav1, anti-EED, anti-H3K27me3 antibodies, respectively.

### ChIP assay

The collected PC 12 cells were cross-linked with 1 % formaldehyde for 10 min at 37°C, washed three times with PBS and lysed with lysis buffer (50mM Hepes-KOH pH7.5; 140mM NaCl; 1mM EDTA; 10 % glycerol; 0.5 % NP-40; 0.25 % Triton X-100). The cross-linked samples were then subjected to sonication for 10 min using Bioruptor (Diagenode, Liege, Belguim). The protocol was set as “Mid” range and 30 seconds “ON” followed by 30 seconds “OFF”, yielding the DNA fragments with the size of 200∼1000 bp. 25 μl of the sonicated samples were pipetted out as input, while 250 μl aliquots were diluted with 555 μl dilution buffer (0.01 % SDS; 1.1 % Triton X-100; 1.2 mM EDTA; 16.7 mM Tris-HCl, pH 8.1; 167 mM NaCl), and then subjected to the subsequent IP experiment. After centrifugation, the soluble fraction was recovered and precleared for 30 min at 4°C with magnetic beads coupled with anti-mouse IgG (Invitrogen, Shanghai, China). Following the removal of the beads, the supernatant was transferred to two new tubes with 425 μl each, and incubated with anti-EZH2 (diluted by 1:500) antibody or unimmune IgG for IP and mock group, respectively. The incubation lasted overnight at 4°C with mild rotation. Chromatin was immunoprecipitated with magnetic beads’ incubation for 1 h at 4°C, followed by the Magnetic Separation Rack’s adsorption for 2 min. After the extensive washing, bound chromatin and input DNA was placed in elution buffer (10 mM Tris-HCl, pH 8.0; 5 mM EDTA; 300 mM NaCl; 0.5 % SDS) and reverse cross-linked. Immunoprecipitated DNA and input DNA were treated with RNaseA (Sigma-Aldrich, Shanghai, China) and proteinase K, prior to being purified with a QIAquick PCR purification kit (QIAGEN, Dusseldorf, Germany). qPCR was performed as indicated previously and primers used were listed in Table 1. % of input was calculated for each ChIP fraction according to the following formula: % Input=2^(Ct Input - Ct ChIP)^ × 10 ×100 %. Normalize each ChIP DNA fractions’ Ct value to that of the input DNA for the same qPCR assay.

### Statistical analysis

Promoter CpG Island prediction was performed using Methprimer software [36]. Statistical tests were performed using one-way analysis of variance (ANOVA) and t-test through SPSS (Version 19.0) software. *, ** and *** were used to represent *P*<0.05, *P*<0.01 and *P*<0.001, respectively.
